# Designing for Comfort in VR Public Speaking: How Avatar Realism and Natural Environments Shape User Experience and Stress Responses

**DOI:** 10.3390/bs16050800

**Published:** 2026-05-17

**Authors:** Han Zhang, Rui Peng, Shiyi Wang, Hanting Song, Zijian Li

**Affiliations:** 1 Intelligent Design, Platform at the Intersection of Art and Technology, Central China Normal University, Wuhan 430079, China; zhanghan120@ccnu.edu.cn (H.Z.); pengrui@mails.ccnu.edu.cn (R.P.); sequins@mails.ccnu.edu.cn (S.W.); 2 Digital Media Art, School of Fine Arts, Central China Normal University, Wuhan 430079, China; songhanting@mails.ccnu.edu.cn

**Keywords:** virtual reality, public speaking, avatar realism, natural environment, social evaluative threat, user experience

## Abstract

Virtual reality (VR) is increasingly used in public speaking training, yet the distinct roles of environmental context and virtual audience design remain unclear. This study examines how avatar visual style (realistic vs. stylized) and scene type (natural vs. indoor) influence subjective experience and physiological stress. A total of 132 participants were assigned to a 2 × 2 between-subjects experiment. Subjective experience was assessed using standardized questionnaires, while physiological responses were measured via electrodermal activity and heart rate variability, complemented by post-experiment interviews. Results revealed a dissociation between subjective and physiological responses. Natural environments significantly enhanced user satisfaction and overall experience, whereas avatar style primarily influenced physiological stress. Specifically, stylized avatars elicited lower electrodermal activity than realistic avatars, indicating reduced sympathetic arousal. No significant interaction effects were observed. Mediation analyses showed no significant roles of perceived support or threat, suggesting that physiological responses may not rely on explicit cognitive appraisal. Qualitative findings further indicated that ambiguous audience feedback limited evaluative interpretation. These findings support a dual-pathway framework in which environmental context shapes cognitive–affective experience, whereas avatar realism modulates implicit physiological stress. This study provides theoretical insights and practical implications for designing VR systems that enhance user comfort and reduce stress.

## 1. Introduction

Virtual reality (VR) has become an increasingly important tool for behavioral training and psychological research due to its ability to create immersive and controllable environments. Compared with traditional screen-based media, VR provides multisensory stimulation and spatial interaction that enhance users’ sense of presence and engagement (Slater & Wilbur, 1997; Slater, 2009). These characteristics allow VR systems to simulate real-world situations while maintaining experimental control, making them valuable platforms for studying human behavior in complex social contexts.

In recent years, immersive VR technologies have been widely applied in education, professional training, and psychological interventions. Research suggests that immersive environments can enhance learning engagement, emotional involvement, and experiential understanding compared with traditional instructional approaches (Makransky & Petersen, 2021). Because VR environments can evoke psychological and physiological responses similar to those occurring in real-world situations, they provide a powerful tool for investigating behavioral responses under controlled yet ecologically valid conditions.

Public speaking represents a particularly relevant domain for VR-based training. Speaking in front of an audience is one of the most common social situations associated with anxiety and stress, largely due to the presence of social evaluative threat—the perception that one’s performance is being judged by others (P. L. Anderson et al., 2013). This form of social evaluation can trigger cognitive stress responses and physiological arousal, which may negatively affect communication performance and learning outcomes. VR-based public speaking systems provide a safe and repeatable environment in which individuals can practice presentations while experiencing simulated audience feedback. Previous studies have shown that VR exposure training can effectively reduce public speaking anxiety and improve communication skills (Pertaub et al., 2002; Ye et al., 2024). More importantly, the effects of VR-based public speaking training may transfer to real-world speaking situations. Prior studies have shown that VR practice can improve subsequent real-life public speaking performance (Kryston et al., 2021; Bachmann et al., 2023). After VR-assisted public speaking training, students’ speeches delivered to a real audience were perceived as more persuasive, and the speakers themselves were perceived as more charismatic (Valls-Ratés et al., 2023). These findings suggest that VR can serve as a controllable rehearsal space that helps users prepare for the demands of real-world public speaking.

However, despite the growing adoption of VR in public speaking training, existing research largely treats VR environments as holistic systems, overlooking how distinct design components may independently and differentially influence user responses. In practice, VR environments are composed of multiple elements, such as environmental context and virtual human characters, each of which may play distinct roles in shaping users’ psychological and behavioral outcomes.

In particular, virtual audience design represents a critical yet underexplored factor. Prior research suggests that users respond to virtual characters as social agents, even when they are aware of their artificial nature (Bailenson et al., 2003). Meanwhile, environmental context may also influence emotional and stress-related responses, as different settings can convey varying levels of social expectation and evaluation pressure (Hartig et al., 2003). In VR public speaking, different scene types may therefore convey varying levels of social expectation and evaluation pressure.

Although previous studies have examined avatar characteristics or environmental features separately, there remains limited understanding of how these design elements jointly—and potentially dissociative—affect subjective experience and physiological stress responses in socially evaluative VR tasks such as public speaking. This gap is particularly important because subjective evaluations and physiological responses do not always align, yet most VR research treats them as convergent indicators of user experience.

To address this limitation, the present study investigates the combined effects of two key design factors—virtual audience visual style (realistic vs. stylized) and VR scene type (natural vs. indoor meeting room)—on user experience and physiological stress responses during VR public speaking tasks. Using a 2 × 2 between-subjects experimental design, this study integrates subjective evaluations with physiological measures, including electrodermal activity (EDA) and heart rate variability (HRV).

This study makes three key contributions. First, it disentangles the independent effects of environmental context and avatar realism in VR public speaking scenarios. Second, it demonstrates a dissociation between subjective experience and physiological stress responses. Third, it proposes a dual-pathway framework suggesting that VR design elements influence users through both cognitive–affective appraisal and implicit physiological mechanisms.

By examining both psychological and physiological responses, this study aims to provide a more comprehensive understanding of how different components of VR environments influence behavior under social evaluation. In doing so, it advances theoretical understanding of social evaluative processes in immersive environments and provides actionable design implications for VR-based training systems.

## 2. Theoretical Framework

### 2.1. Social Evaluative Threat

Social evaluative threat refers to situations in which individuals perceive that they are being judged by others, particularly in contexts where their performance may be evaluated against social standards. This form of threat is a central mechanism underlying stress responses in social situations such as public speaking (Poppelaars et al., 2019), job interviews (Bosshard et al., 2023), and performance assessments (Craw et al., 2021). According to social self-preservation theory, threats to one’s social image or status can trigger both psychological stress and physiological activation, including increased sympathetic nervous system activity (Dickerson & Kemeny, 2004).

Public speaking is widely recognized as a prototypical context of social evaluative threat. Empirical research has consistently shown that speaking in front of an audience induces both subjective anxiety and physiological arousal, including elevated skin conductance and altered heart rate variability (P. L. Anderson et al., 2013; Allen et al., 2017; Poppelaars et al., 2019). These responses are primarily driven by perceived evaluation rather than the task itself, highlighting the central role of social context in shaping stress reactions.

Importantly, such social evaluative processes are not limited to real-world interactions. Studies in virtual environments demonstrate that individuals respond to virtual audiences as if they were real social agents, exhibiting comparable behavioral and physiological reactions (Slater et al., 2006; Kroczek & Mühlberger, 2023). This suggests that virtual audiences can effectively elicit social evaluative threat when they are perceived as meaningful observers.

However, in virtual reality (VR)-based public speaking training, the intensity of social-evaluative threat may depend on the specific training objectives and the stage of intervention. Previous research on VR experiences has suggested that virtual social threat can be graded through factors such as audience size, audience responses, and self-salience (Premkumar et al., 2021). This suggests that, for speakers with elevated levels of public speaking anxiety, VR training environments can be carefully designed to elicit anxiety-related experiences that partially simulate real-life public speaking situations, thereby enabling users to identify, regulate, and gradually adapt to audience-related evaluative pressure within a relatively controlled setting.

Within immersive VR environments, social evaluative threat is primarily conveyed through virtual human characters. Previous VR public speaking studies have shown that virtual audiences can elicit anxiety responses and that the type of audience feedback influences the intensity of speakers’ anxiety (Pertaub et al., 2002). More recent work further suggests that audience-related factors, including audience support, audience engagement, audience size, and room dimensions, may shape public speaking anxiety in virtual scenarios (Kroczek & Mühlberger, 2023; Ye et al., 2024).The extent to which users perceive these characters as credible observers influences the intensity of the threat response. Research on social presence suggests that even minimal social cues in virtual agents can trigger automatic social cognition processes (Vanni et al., 2013; Klein, 2025). Importantly, social evaluative threat involves both conscious appraisal (e.g., feeling judged or threatened), and automatic physiological activation (e.g., sympathetic arousal and cardiovascular stress responses) (Tomaka et al., 1993; Bosch et al., 2009; Woody et al., 2018).This suggests that different aspects of VR design may influence subjective appraisal and physiological activation through partially distinct pathways.

### 2.2. Avatar Realism

Virtual human characters serve as key carriers of social information in immersive environments. Their appearance, behavior, and level of realism shape users’ perceptions of social presence and interaction. According to anthropomorphism theory, individuals tend to attribute human-like intentions, emotions, and agency to entities that possess human-like features (Epley et al., 2007). As a result, more human-like avatars are generally associated with stronger perceptions of social presence and interpersonal relevance (Nowak & Biocca, 2003).

However, increasing realism does not always lead to more positive user experiences. The uncanny valley hypothesis proposes that entities that are highly human-like but not perfectly realistic can evoke discomfort, eeriness, or even threat (MacDorman & Ishiguro, 2006; Mori et al., 2012). Empirical studies have provided support for this phenomenon, showing that near-realistic virtual characters can elicit negative affective responses and reduce user acceptance (Seyama & Nagayama, 2007).

In VR contexts, where immersion and presence are heightened, the effects of avatar realism may be particularly pronounced. Research indicates that highly realistic avatars can increase users’ sense of being observed, thereby intensifying social pressure in evaluative tasks (Bailenson et al., 2003; Pan & Hamilton, 2018). Conversely, stylized or cartoon-like avatars may reduce perceived social threat by signaling a lower level of realism and evaluation relevance (Zhu & Broadbent, 2025; Dubosc et al., 2025; Takemoto & Sugiura, 2026). However, audience stance and behavioral cues also shape perceived social evaluation, negative audience behaviors, such as distraction, lack of eye contact, interruptions, or background noise, can intensify speakers’ anxiety and affect speech production in VR public speaking tasks (Rodero & Larrea, 2022).

Crucially, avatar realism may operate at a perceptual and implicit level, directly modulating users’ sense of being observed and triggering physiological stress responses even in the absence of explicit evaluative judgments. This effect should therefore be understood alongside other audience-related cues, including behavioral and attitudinal signals, which may further amplify or reduce perceived evaluative threat. Drawing on social presence theory, avatar realism may strengthen users’ perception that they are being observed by socially meaningful others, thereby increasing the salience of evaluative cues in the VR public speaking task (Oh et al., 2018). From a social–psychophysiological perspective, the presence of an audience can elicit challenge- or threat-related physiological responses during performance situations (Blascovich et al., 1999). Therefore, avatar realism is expected to be more closely associated with implicit physiological stress responses, although it may also influence subjective experience.

Taken together, avatar realism and audience-related behavioral cues are likely to influence how strongly virtual audiences are perceived as evaluative agents, thereby shaping users’ emotional and physiological responses in VR public speaking contexts. In the present study, we focus specifically on avatar visual realism while acknowledging that audience behavior and stance constitute important additional factors in VR public speaking design.

### 2.3. Restorative Environment

Environmental context plays a crucial role in shaping emotional and physiological responses. According to Attention Restoration Theory (ART), natural environments facilitate cognitive recovery by providing stimuli that are softly engaging and require minimal directed attention (Kaplan, 1995). Similarly, Stress Recovery Theory (SRT) posits that exposure to natural environments can rapidly reduce physiological stress and promote positive affect (Ulrich et al., 1991).

A substantial body of research has demonstrated the restorative effects of natural environments on both psychological and physiological outcomes. For example, exposure to natural scenes has been associated with reduced stress, improved mood, and lower physiological arousal, including decreased skin conductance and increased heart rate variability (Berto, 2005; Jimenez et al., 2021; Koivisto & Grassini, 2024). Importantly, these effects have also been observed in virtual environments. Studies have shown that virtual nature can produce comparable restorative benefits, suggesting that the psychological mechanisms underlying restoration extend to simulated environments (C. A. Anderson et al., 2017; Browning et al., 2020; Spano et al., 2023; Chou et al., 2026).

In contrast, built environments such as offices, classrooms, and meeting rooms are often associated with task demands and social expectations (Ulrich, 1984; Ewert & Chang, 2018; Hao et al., 2024). Such environments may function as high-pressure contexts that heighten perceived evaluation and cognitive load. In VR public speaking scenarios, an indoor meeting room may be interpreted as a formal evaluative setting, thereby enhancing social evaluative threat. Whether a virtual environment should intensify or attenuate social evaluative pressure depends on the intended purpose of the VR public speaking system.

Furthermore, environmental context may influence how individuals interpret social cues. Research suggests that supportive or relaxing environments can attenuate stress responses and reduce sensitivity to negative stimuli (Ewert & Chang, 2018). Conversely, formal or high-demand environments may amplify the impact of social evaluative cues. Therefore, environmental context is expected to operate primarily through cognitive–affective appraisal rather than through immediate social-observational threat. Environmental psychology suggests that physical and simulated environments can shape users’ affective evaluations, such as pleasantness, arousal, comfort, and approach-related responses (Russell & Pratt, 1980). In the present study, the natural and indoor meeting-room environments were therefore conceptualized as contextual frames that may influence overall user experience, perceived comfort, and appraisal of the speaking situation. We propose that its dominant pathway is more likely to involve conscious appraisal and affective evaluation.

In the present study, the comparison between natural and indoor environments allowed us to examine how different environmental contexts shape users’ subjective appraisal, affective experience, and physiological stress responses during VR public speaking.

### 2.4. Integrative Perspective: A Dual-Pathway Framework

Taken together, these perspectives suggest that VR public speaking experiences may be shaped by two partially distinct but not mutually exclusive pathways. In the present framework, avatar realism is expected to operate through perceptual responses: highly realistic virtual audiences may enhance social presence and the sense of being observed, thereby triggering implicit physiological stress responses in socially evaluative performance tasks. Environmental context is expected to operate through a cognitive–affective appraisal pathway. A natural environment may improve subjective experience by providing a more restorative or supportive contextual frame, whereas an indoor meeting room may convey stronger formality and evaluative expectations.

This distinction implies a potential dissociation between subjective experience and physiological stress responses, such that users may report positive experiences while still exhibiting elevated physiological arousal, or vice versa.

Accordingly, this study proposes a dual-pathway framework in which (1) environmental context shapes subjective experience through conscious appraisal processes, whereas (2) avatar realism influences physiological stress through implicit perceptual mechanisms. This framework provides the theoretical basis for examining the independent and combined effects of VR design elements in socially evaluative contexts.

## 3. Hypotheses

### 3.1. Effects of Scene Type on User Experience

Environmental context has been shown to influence individuals’ emotional states and subjective evaluations, with natural environments generally associated with lower stress and more positive experiences (Ulrich et al., 1991; Kaplan, 1995). In contrast, formal indoor environments may increase perceived evaluation pressure in public speaking contexts. From a cognitive–affective perspective, environmental context is expected to shape how users consciously appraise and evaluate their experience within VR. Given these differences, VR scene type is expected to influence users’ subjective experience during public speaking tasks.

**H1.**  *Compared with indoor meeting room environments, natural VR environments will lead to more positive subjective user experience, including higher satisfaction and overall experience evaluation.*

### 3.2. Effects of Avatar Realism on Physiological Stress Responses

Virtual audiences serve as primary sources of social evaluative cues in VR public speaking scenarios. Prior research suggests that higher levels of human likeness can increase perceived observation and evaluation, thereby intensifying stress responses (Pan & Hamilton, 2018). From an implicit processing perspective, avatar realism may directly modulate physiological stress responses by influencing perceived social presence and evaluative salience, even in the absence of explicit cognitive appraisal. Accordingly, avatar realism is expected to influence users’ physiological stress responses during VR public speaking tasks.

**H2.**  *Compared with realistic virtual audiences, stylized virtual audiences will elicit lower physiological stress responses, as reflected in reduced electrodermal activity and more favorable heart rate variability.*

### 3.3. Relationship Between Scene Type and Avatar Realism

Environmental context may also influence how individuals respond to social evaluative cues. Research suggests that restorative environments can attenuate stress responses, whereas formal environments may heighten sensitivity to evaluation (Ewert & Chang, 2018). However, based on the proposed dual-pathway framework, environmental context and avatar realism may operate through partially independent mechanisms rather than producing strong interaction effects.

**H3.**  *Scene type and avatar realism are expected to exert largely independent effects on user experience and physiological stress responses, with limited or non-significant interaction effects.*

## 4. Methods

### 4.1. Participants

A priori power analysis was conducted using G*Power 3.1, indicating that a minimum sample size of 128 would be sufficient to detect medium effect sizes (f = 0.25) with 80% power at α = 0.05. Participants were recruited on a voluntary basis through university-based recruitment channels, including campus advertisements and online student communication groups. A total of 132 participants, including 68 undergraduate students (51.5%) and 64 graduate students (48.5%). The sample consisted of 73.5% females and 26.5% males, with a mean age of 21.72 years (range = 18–36). The relatively high proportion of female participants partly reflected the recruitment context, as participants were recruited from a normal university with a higher proportion of female students. All participants reported no history of severe psychological disorders and no adverse reactions to VR exposure. Participants were also required to have basic public speaking ability to complete the experimental tasks.

All participants provided informed consent prior to the experiment and were fully briefed about the procedure. Prior VR experience or public speaking experience may influence participants’ responses to VR public speaking tasks, these experience-related differences were considered potential sources of individual variation. However, these factors were not the primary variables of interest in the present study. Participants were randomly assigned to one of the four experimental conditions to reduce systematic bias across groups (*n* = 33 per group). Participants received compensation for their participation in accordance with institutional guidelines.

### 4.2. Design and Procedure

The study employed a 2 (scene type: natural vs. indoor meeting room) × 2 (avatar style: realistic vs. stylized) between-subjects experimental design.

Before the experiment, participants were informed that they would complete two VR public speaking tasks and that their questionnaire responses and physiological signals would be recorded throughout the experiment. They read the experimental instructions before entering the VR tasks. To reduce demand characteristics, the specific hypotheses concerning scene type and avatar realism were not disclosed prior to the tasks. They were then equipped with a VR headset and physiological recording devices.

**Figure 1:**
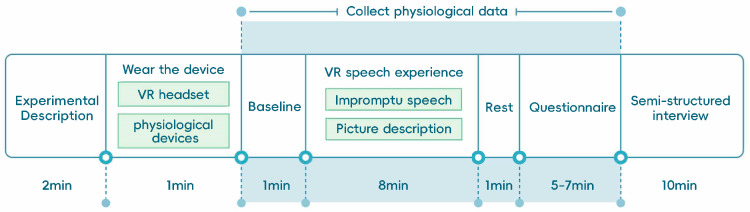
Experimental procedure and timeline.

The experimental procedure consisted of five sequential phases: adaptation, baseline recording, VR speech tasks, questionnaires, and a semi-structured interview (see Figure 1). First, participants completed an adaptation phase (~2 min) to familiarize themselves with the VR environment and reduce potential novelty or simulator effects. Next, a baseline phase (1 min) was conducted, during which participants remained in the virtual environment without performing any task, allowing for stabilization of physiological signals. Participants then engaged in the VR speech task phase (~8 min), which consisted of two speaking tasks: (1) an impromptu speech based on a given topic, and (2) a picture-based descriptive speech (see Appendix A.1 and Appendix A.2).

Each task lasted approximately 4 min, with 30 s of preparation time provided before each task. Virtual audiences were presented according to the assigned experimental condition, and physiological data were continuously recorded throughout this phase. Following the speech tasks, participants completed a brief rest period (1 min) to allow physiological responses to return toward baseline.

Participants then completed a set of post-experiment questionnaires (approximately 5–7 min) assessing user experience, usability, and audience perception.

Finally, participants took part in a semi-structured interview (approximately 10 min) designed to capture their subjective experiences and perceptions of the VR environment. The interview protocol is provided in Appendix Semi-Structured Interview Questions.

All sessions were audio- and video-recorded for consistency and subsequent analysis.

### 4.3. Materials and Apparatus

The virtual environments were developed using Unreal Engine 5.1. The experiment was conducted using an HTC Vive Pro head-mounted display (manufacturer: HTC Corporation, Taichung, China; resolution: 1440 × 1600 pixels per eye; refresh rate: 90 Hz), which provided an immersive visual experience. Physiological data, including heart rate and electrodermal activity (EDA), were recorded using a wearable device (Huixin wristband;manufacturer: Huixin Technology Co., Ltd., Shenzhen, China) synchronized with the VR system. EDA signals were recorded at a sampling rate of 250 Hz and preprocessed using standard filtering procedures to remove noise and motion artifacts.

**Figure 2:**
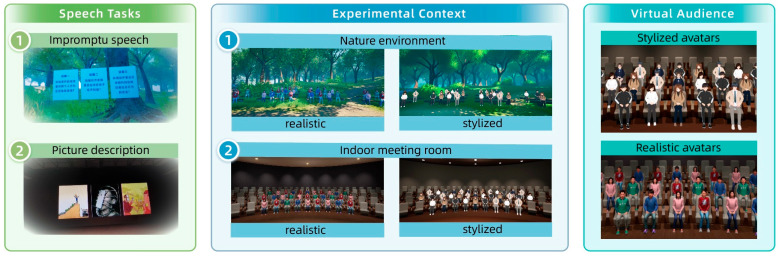
Experimental tasks and VR conditions used in the study. (**Left**) Speech tasks used in the experiment, including an impromptu speech task (an English translation of the full text is provided in Appendix A.1) and a picture description task. (**Middle**) Environmental context conditions presented in the VR scenarios, including natural and indoor meeting-room environments combined with different avatar styles. (**Right**) Two styles of virtual audiences used in the experiment: stylized avatars and realistic avatars.

As shown in Figure 2, two types of virtual environments were constructed: a natural outdoor scene and an indoor meeting room. Both environments were controlled to ensure consistency in lighting conditions, sound settings, spatial scale, and viewing perspective, differing only in environmental context. The virtual audience consisted of 35 characters seated in front of the participant. All avatars displayed neutral sitting postures with basic idle movements and maintained gaze toward the speaker. Two visual styles were designed: realistic avatars with detailed human-like appearance, and stylized avatars with simplified, cartoon-like features.

Across conditions, audience size, spatial arrangement, gaze behavior, and animation were held constant, with only visual style manipulated.

### 4.4. Measures

Subjective responses were assessed using standardized questionnaires. User satisfaction (US) was measured using the Satisfaction Assessment Questionnaire (SEQ), while perceived usability (TU)was assessed using items adapted from the System Usability Scale (SUS) (Hyzy et al., 2022) and the User Experience Questionnaire (UEQ) (Schrepp et al., 2017). In the present study, user experience was operationalized as a composite index based on these two components. Specifically, the user experience score was calculated as the mean of the US and TU composite scores. Audience perception (support and threat) was measured using the Perceived Audience Scale (Salminen et al., 2020). All items were rated on a 5-point Likert scale, the details see Appendix A.3. Composite scores were calculated by averaging item responses for each construct.

In addition to the questionnaire-based measures, post-experimental interviews were conducted as a complementary qualitative method to provide contextual explanations for participants’ subjective responses to the VR public speaking task. The interview questions mainly focused on participants’ perceptions of the realism or virtuality of the VR environment, the influence of the environment on attention and emotion, possible sources of distraction, perceived support or threat from the virtual audience, the influence of audience reactions on attention and stress, the role of visual style in shaping the speaking experience, differences between anxiety in VR and anxiety in front of a real audience, emotion-regulation strategies during the speech, task difficulty and cognitive load, and suggestions for improving the VR public speaking environment. These interviews helped us further understand how participants interpreted the virtual audience and environmental context across different experimental conditions.

Physiological stress responses were assessed using electrodermal activity (GSR) and heart rate variability (HRV). GSR was recorded at a sampling rate of 250 Hz. HRV analysis was conducted using Kubios HRV Premium software 3.5.0 with automatic artifact correction (medium threshold), following established psychophysiological guidelines (Quigley et al., 2024). Both time-domain (SDNN, RMSSD) and frequency-domain (LF, HF, LF/HF ratio) indices were calculated. Respiration was not directly controlled in this study, which may influence HRV indices; therefore, HRV results should be interpreted with caution.

### 4.5. Data Processing and Analysis

Physiological data were processed by calculating change scores (Δ = task phase − baseline) for each participant. HRV data were standardized prior to analysis to reduce inter-individual variability. Statistical analyses were conducted using Python (version 3.12.7). Two-way between-subjects ANOVAs were used to examine the main and interaction effects of scene type and avatar style. Prior to analysis, assumptions of normality and homogeneity of variance were assessed using Shapiro–Wilk tests and Levene’s tests, respectively. To examine potential gender effects, a three-way between-subjects ANOVA (scene type × avatar style × gender) was conducted on physiological responses. Mediation analyses were conducted only for pathways that met theoretical expectations and statistical preconditions, including a significant association between the independent variable and the dependent variable.

To further examine indirect effects, bootstrap mediation analysis with 5000 resamples was performed. Statistical significance was evaluated at α = 0.05. Where applicable, false discovery rate (FDR) correction was applied to control for multiple comparisons.

Reliability analyses indicated that all subjective measures had satisfactory internal consistency, with Cronbach’s α values exceeding 0.75. Exploratory factor analysis was performed to assess the construct validity of the subjective measures, and Kaiser-Meyer-Olkin (KMO) values for all scales exceeded 0.75, with significant Bartlett’s tests of sphericity (*p* < 0.001), confirming the appropriateness of the data for subsequent analyses.

## 5. Results

### 5.1. Descriptive Statistics

Descriptive statistics for all subjective and physiological measures across experimental conditions (natural vs. indoor environments, stylized vs. realistic avatars) are summarized in Table 1. Table 1 presents the means (M) and standard deviations (SD) of subjective measures (user experience, satisfaction, usability, perceived support, and perceived threat) and physiological indices (ΔEDA and ΔRMSSD) across the four experimental conditions.

Preliminary inspection indicated that participants in natural environments generally reported higher subjective ratings, whereas stylized avatars were associated with lower electrodermal activity, suggesting reduced physiological arousal.

### 5.2. Effects on Subjective Experience

#### 5.2.1. User Experience and Satisfaction

**Figure 3:**
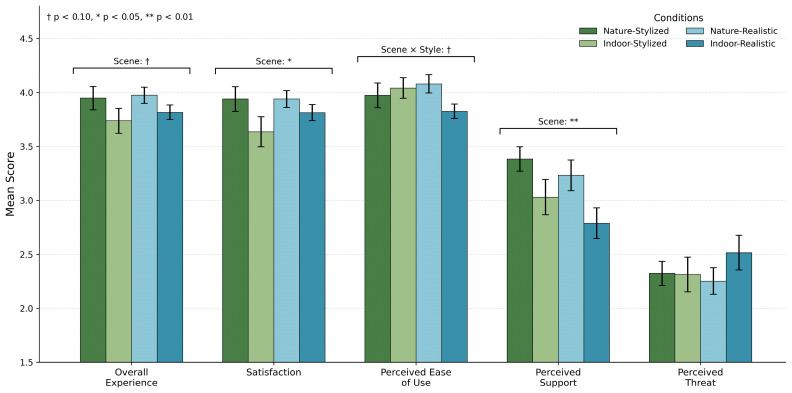
Subjective ratings across four VR conditions. Bars represent mean scores (± SE) for overall experience, satisfaction, perceived ease of use, perceived audience support, and perceived threat in the Nature–Stylized, Indoor–Stylized, Nature–Realistic, and Indoor–Realistic conditions.

Two-way between-subjects ANOVAs were conducted to assess the effects of scene type (natural vs. indoor) and avatar style (realistic vs. stylized) on user experience (UX) and satisfaction (see Figure 3). For overall UX, the analysis revealed a marginal main effect of scene type, F (1, 128) = 3.85, *p* = 0.052, ηp^2^ = 0.029, indicating a small effect size. Participants in the natural condition (M = 3.96, SD = 0.72) reported slightly higher UX than those in the indoor condition (M = 3.78, SD = 0.74). No significant main effect of avatar style or interaction effect was observed (*p* > 0.05).

For user satisfaction (US), a significant main effect of scene type was found, F (1, 128) = 4.16, *p* = 0.043, ηp^2^ = 0.031, indicating a small effect size. Participants reported higher satisfaction in the natural environment (M = 3.84, SD = 0.79) compared to the indoor setting (M = 3.62, SD = 0.80). No significant interaction effects were observed, consistent with the assumption of independent effects of scene context and avatar design.

#### 5.2.2. Usability

For usability (TU), neither the main effects of scene type nor avatar style were significant (*p* > 0.05). However, the interaction effect approached significance, F (1, 128) = 3.07, *p* = 0.082, ηp^2^ = 0.023, indicating a small effect size. This trend suggests that stylized avatars may be associated with slightly higher usability ratings in indoor environments.

#### 5.2.3. Audience Perception

For perceived audience support, a significant main effect of scene type was found, F (1, 128) = 7.99, *p* = 0.006, ηp^2^ = 0.059, indicating a small-to-medium effect size. (Participants in the natural condition reported higher perceived support (M = 3.92, SD = 0.68) than those in the indoor condition (M = 3.55, SD = 0.70). No significant main effects or interaction effects were found for perceived threat, with all *p*-values exceeding 0.05.

### 5.3. Effects on Physiological Responses

**Figure 4:**
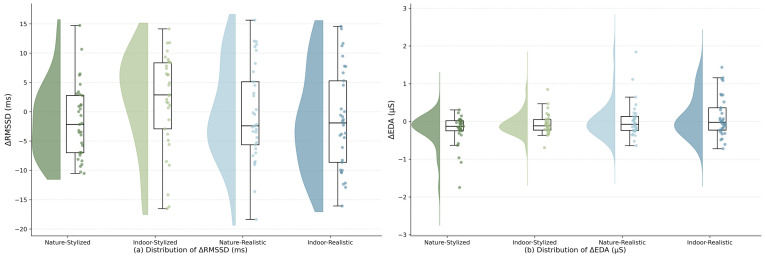
Raincloud plots showing the distribution of physiological changes across four VR conditions (Nature-Stylized, Indoor-Stylized, Nature-Realistic, and Indoor-Realistic). (**a**) ΔRMSSD (ms), reflecting parasympathetic activity; a marginal interaction effect was observed. (**b**) ΔEDA (μS), reflecting sympathetic arousal; a significant main effect of avatar style was found. Plots include data distribution, jittered individual observations, and corresponding boxplots.

To assess physiological stress responses, two-way ANOVAs were conducted on ΔEDA and ΔRMSSD, calculated as the change from baseline (T2 − T1), as shown in Figure 4.

For heart rate variability, no significant main effects of scene type, F (1, 128) = 0.86, *p* = 0.355, or avatar style, F (1, 128) = 0.59, *p* = 0.445, were observed. However, the interaction effect approached significance, F (1, 128) = 3.11, *p* = 0.080, ηp^2^ = 0.023, indicating a small effect size. In the indoor condition, stylized avatars were associated with a positive shift in ΔRMSSD (M = 1.97 ms), whereas realistic avatars showed a decrease (M = −1.46 ms). In contrast, differences were minimal in the natural condition.

For electrodermal activity (ΔEDA), a significant main effect of avatar style was found, F (1, 128) = 7.92, *p* = 0.006, ηp^2^ = 0.058, indicating a small-to-medium effect size.

Consistent with the descriptive statistics in Table 1, participants in the stylized avatar conditions (M ≈ 0.55) exhibited lower ΔEDA than those in the realistic conditions (M ≈ 0.73), indicating reduced sympathetic arousal. No significant main effect of scene type (*p* = 0.104) or interaction effect (*p* = 0.781) was observed. These results suggest that physiological stress responses were primarily influenced by avatar realism rather than environmental context.

Additional analyses including gender as a moderator revealed no significant main effect of gender on ΔEDA, F (1, 124) ≈ 0.00, *p* = 0.999.

Furthermore, no significant interaction effects involving gender were observed, including scene × gender, F (1, 124) = 1.47, *p* = 0.227, avatar × gender, F (1, 124) = 0.99, *p* = 0.323, and the three-way interaction, F (1, 124) = 0.36, *p* = 0.547. To further verify the robustness of the findings, additional regression analyses were conducted, yielding consistent results.

### 5.4. Mediation Analysis

To examine whether perceived audience support and perceived threat mediate the effects of avatar style on user experience and physiological stress, two separate mediation models were tested using bootstrap procedures with 5000 resamples. The results are summarized in Table 2.

For user experience, perceived support significantly predicted UX (b = 0.285, 95% CI [0.181, 0.389]), but avatar style did not significantly predict perceived support (a = 0.197, 95% CI [−0.081, 0.475]). The indirect effect was not significant (95% CI included zero), indicating no mediation effect. For physiological stress (ΔEDA), avatar style showed a significant total effect (c = −0.212, 95% CI [−0.361, −0.064]), whereas perceived threat did not significantly predict ΔEDA. The indirect effect was not significant.

Importantly, the absence of significant mediation suggests that the effect of avatar realism on physiological stress may not depend on explicit cognitive appraisal (e.g., perceived support or threat), but may instead reflect more direct or implicit processes.

In addition to the hypothesized mediation models, an exploratory mediation analysis was conducted to examine whether perceived audience support mediates the relationship between scene type and user experience.

The results showed that scene type significantly predicted perceived support (β = 0.399, *p* = 0.005), and perceived support significantly predicted user experience (β = 0.285, *p* < 0.001). The total effect of scene type on user experience was marginally significant (β = 0.184, *p* = 0.051), while the direct effect became non-significant after including perceived support (β = 0.071, *p* = 0.419). These results suggest that perceived support fully mediates the relationship between scene type and user experience.

**Figure 5:**
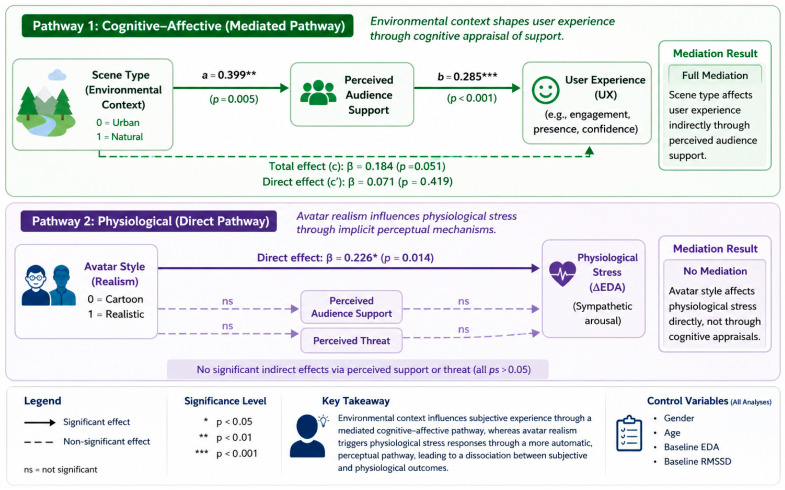
Dual-pathway model illustrating the dissociation between cognitive–affective and physiological responses in VR public speaking. Environmental context (scene type) affects user experience (UX) through perceived audience support (mediated pathway), whereas avatar realism influences physiological stress responses (ΔEDA) directly without mediation.

The overall pattern of results is summarized in Figure 5, illustrating a dual-pathway model in which environmental context influences user experience through a mediated cognitive–affective pathway, whereas avatar realism directly affects physiological stress responses.

### 5.5. Qualitative Insights from Post-Experiment Interviews

To further interpret the quantitative findings, semi-structured interviews were conducted following the experiment. Using thematic analysis in psychology was used to identify recurring patterns in participants’ experiences across different VR conditions, see Table 3.

Many participants reported that realistic virtual audiences heightened their sense of being observed and evaluated. The human-like appearance and gaze behavior of realistic avatars led participants to interpret them as socially relevant agents, thereby increasing performance pressure. Participants frequently described the experience as “similar to speaking in front of real people” or noted that they felt “watched” during the task. This aligns with the concept of social evaluative threat, suggesting that higher avatar realism strengthens perceived evaluation cues.

In contrast, stylized avatars were often perceived as less intimidating and more approachable. Participants reported feeling more relaxed and less judged when interacting with stylized audiences. Some participants explicitly noted that stylized avatars made the task feel “less serious” or “more like a simulation,” which reduced anxiety and allowed them to focus more on the content of their speech. This pattern is consistent with the reduced ΔEDA observed in the stylized conditions.

For natural environments that facilitate emotional regulation, participants consistently reported that natural environments (e.g., outdoor or nature-based scenes) promoted a sense of calmness and reduced tension compared to indoor meeting-room settings. Natural scenes were described as “soothing” and “less formal,” which helped participants regulate their emotions during the speaking task. In contrast, indoor environments were often associated with formal evaluation contexts (e.g., classrooms or meetings), leading to increased perceived pressure. This finding aligns with the higher subjective ratings observed in natural conditions.

Participants consistently reported that natural environments (e.g., outdoor or nature-based scenes) promoted a sense of calmness and reduced tension compared to indoor meeting-room settings. Natural scenes were described as “soothing” and “less formal,” which helped participants regulate their emotions during the speaking task. In contrast, indoor environments were often associated with formal evaluation contexts (e.g., classrooms or meetings), leading to increased perceived pressure. This may help explain the absence of significant mediation effects in the quantitative analysis.

## 6. Discussion

The present study examined how virtual environment context and avatar style jointly influence user experience and physiological stress responses in VR public speaking scenarios. By integrating subjective evaluations, physiological measures, and qualitative insights, the findings reveal a clear dissociation between cognitive–affective experience and physiological reactivity.

This study makes an important theoretical contribution by demonstrating that subjective experience and physiological stress responses in VR do not necessarily converge, but can be shaped by distinct underlying mechanisms.

Overall, three key patterns emerged. First, scene type significantly influenced subjective experience, with natural environments consistently associated with higher satisfaction and overall user experience. Second, avatar style primarily affected physiological responses, as stylized audiences elicited significantly lower electrodermal activity compared to realistic audiences. Third, the interaction between scene type and avatar style was not statistically significant, supporting the notion that these two design factors operate through relatively independent pathways rather than synergistic effects.

Taken together, these findings support a dual-pathway framework, in which environmental context primarily influences conscious evaluation and affective appraisal, whereas avatar realism modulates implicit physiological stress responses. This distinction extends existing theories of social evaluative threat by showing that evaluative cues in VR can differentially affect explicit experience and implicit physiological activation.

### 6.1. Effects of Scene Context on Subjective Experience (H1)

Consistent with H1, natural environments led to significantly higher user satisfaction and marginally higher overall user experience compared to indoor meeting room settings. This finding aligns with established theories in environmental psychology, particularly Attention Restoration Theory (Kaplan, 1995) and Stress Reduction Theory (Ulrich, 1984), which posit that exposure to natural environments facilitates emotional recovery and reduces cognitive fatigue. Recent VR research further supports this view, showing that virtual nature environments enhance well-being, reduce perceived stress, and improve engagement (Browning et al., 2020; Chirico & Gaggioli, 2023).

In the context of VR public speaking, natural environments may attenuate perceived social pressure by reducing the formality and evaluative connotations associated with indoor meeting spaces. This interpretation is supported by qualitative reports, in which participants described natural scenes as more “relaxing” and less “judgmental,” whereas indoor environments were frequently associated with evaluation and performance pressure. Notably, the effect of scene type was more pronounced for satisfaction and perceived support than for usability, suggesting that environmental context primarily influences affective and experiential dimensions rather than functional judgments. This pattern is consistent with prior HCI research indicating that contextual and aesthetic factors exert stronger effects on emotional appraisal than on perceived usability (Hassenzahl & Monk, 2010).

Importantly, these findings support the proposed cognitive–affective pathway, indicating that environmental context primarily shapes how users consciously interpret and evaluate their experience rather than directly modulating physiological stress responses.

### 6.2. Effects of Avatar Style on Psychological Responses (H2)

The results provide clear support for H2, demonstrating that stylized avatars elicited significantly lower ΔEDA compared to realistic avatars, indicating reduced sympathetic arousal. This finding is consistent with prior research on social evaluative threat, which suggests that more human-like or realistic virtual agents intensify perceived social evaluation and increase physiological stress responses (Blascovich et al., 2002; Dickerson & Kemeny, 2004).

From a theoretical perspective, this effect can be understood through the lens of social presence and anthropomorphism. While realistic avatars enhance perceived social presence, they may simultaneously increase evaluative pressure, leading to heightened physiological arousal. Stylized avatars, by reducing perceptual realism, appear to weaken this evaluative signal and create a psychologically safer interaction context. This interpretation is consistent with recent findings showing that less anthropomorphic agents are often perceived as more approachable and less threatening in emotionally sensitive contexts (Kaiser et al., 2025; Lim et al., 2025). Crucially, these findings suggest that avatar realism operates at an implicit level, directly influencing physiological stress responses without necessarily requiring conscious appraisal.

Interestingly, heart rate variability (ΔRMSSD) did not show significant main effects, suggesting that avatar style primarily influences acute sympathetic activation rather than parasympathetic regulation. This divergence highlights the importance of distinguishing between different physiological systems. While EDA is highly sensitive to immediate arousal and stress responses, HRV reflects more complex regulatory processes that may be influenced by cognitive load (Kim et al., 2018; Fawwaz et al., 2024), breathing patterns (Alshanskaia et al., 2024; Dybvik et al., 2025), and task engagement (Schneider et al., 2025). Thus, the results indicate that avatar realism selectively affects specific components of physiological stress, further supporting the notion of differentiated response pathways.

### 6.3. Interaction Effects and Underlying Mechanisms (H3)

Contrary to traditional expectations of interaction effects, the results showed no significant interaction between scene type and avatar style. Rather than representing a null finding, this pattern provides empirical support for the proposed dual-pathway framework.

Specifically, environmental context and avatar realism appear to operate at different levels of processing. Scene type primarily shapes contextual framing and emotional atmosphere, influencing how users consciously interpret and evaluate the interaction. In contrast, avatar realism directly affects social perception and evaluative salience, triggering more automatic physiological responses.

Another key insight comes from the mediation analysis, which showed that neither perceived support nor perceived threat mediated the relationship between avatar style and user outcomes. This suggests that physiological responses to avatar realism may bypass explicit cognitive appraisal processes. This interpretation is consistent with dual-process theories of emotion, which distinguish between automatic affective reactions and conscious evaluations (Mauss & Robinson, 2009).

Importantly, additional analyses revealed that perceived audience support fully mediated the relationship between scene type and user experience. This finding suggests that the effect of environmental context operates through cognitive–affective appraisal processes, specifically through enhancing perceived social support.

In contrast, no mediation effects were observed for avatar realism, indicating that physiological responses may be driven by more implicit perceptual mechanisms. Together, these findings provide strong support for the proposed dual-pathway framework.

Qualitative findings further support this mechanism. Participants frequently reported ambiguity in audience feedback, indicating difficulty in forming clear evaluative judgments. This ambiguity likely weakened cognitive mediation pathways, while physiological responses remained sensitive to perceptual realism.

Additional analyses further indicated that gender did not significantly moderate the observed effects. This suggests that the influence of avatar realism on physiological stress responses may be robust across genders, providing additional support for the proposed dual-pathway framework. However, given the gender imbalance in the sample, these findings should be interpreted with caution, and future research should examine potential gender differences using more balanced samples.

Taken together, these findings suggest that avatar realism influences physiological stress through implicit perceptual mechanisms, whereas environmental context shapes subjective experience through conscious appraisal processes, leading to a dissociation between subjective and physiological outcomes.

### 6.4. Limitation and Future Directions

Several limitations of the present study should be acknowledged, which also point to directions for future research.

First, the sample consisted primarily of university students, which may limit the generalizability of the findings. Future studies should include more diverse populations, particularly individuals with higher levels of public speaking anxiety or professional experience. The sample also showed a gender imbalance, with a higher proportion of female participants. Although this partly reflected the demographic composition of the recruitment context, it may limit the generalizability of the findings. Future studies could recruit more gender-balanced samples.

Second, the virtual audience behavior was relatively static, with limited expressive feedback. While this ensured experimental control, it may have reduced the clarity of social cues and contributed to the absence of mediation effects. Future research should incorporate more dynamic and responsive audience behaviors to better simulate social interaction.

Third, physiological measures were based on short-term responses during brief speaking tasks. Although electrodermal activity and heart rate variability are widely used indicators of stress, they capture only specific aspects of autonomic activity. In addition, the study did not include a brief self-assessed state anxiety rating immediately before the speech, which limits direct comparison between anticipatory subjective anxiety, physiological arousal, and post-task evaluations. Future studies could include brief state anxiety ratings before and after the task.

Additionally, respiration was not controlled in the present study, which may have influenced HRV indices. Future research should incorporate respiratory monitoring or additional physiological measures (e.g., cortisol) to provide a more comprehensive assessment of stress responses.

Finally, future studies could examine the longitudinal effects of repeated VR training to determine whether the observed dissociation between subjective experience and physiological stress persists over time or changes with adaptation. Future research could also manipulate audience size as an additional design variable to examine how the number of virtual observers shapes perceived evaluative pressure and physiological arousal. Future work could also incorporate an embedded scoring mechanism to assess speech quality, such as fluency, clarity, persuasiveness, confidence, and overall performance. This would allow researchers to further examine how subjective experience and physiological stress responses are related to actual public speaking performance during repeated VR training.

### 6.5. Design Implications

The findings provide several practical implications for the design of VR public speaking systems.

First, avatar style should be selected according to the intended training goal and user group. Stylized avatars may be more suitable for novice users, high-anxiety speakers, or early-stage training, where reducing excessive stress and improving comfort are important. In contrast, realistic avatars may be more appropriate for advanced training or assessment contexts, where simulating real-life evaluative pressure is desirable. This recommendation is consistent with social presence theory, which suggests that more socially realistic agents may enhance the perceived presence of others, but also increase the salience of being observed and evaluated (Blascovich et al., 1999; Oh et al., 2018).

In addition, environmental context should be treated as a contextual design tool rather than a neutral background in VR public speaking systems. When the goal is to improve subjective experience and reduce the perceived formality of the task, natural environments may be more useful. Conversely, when the goal is to simulate formal evaluative situations, an indoor meeting-room environment may be more appropriate. This implication is consistent with environmental psychology and VR exposure research, which suggest that environments can shape affective appraisal and that virtual social threat can be graded according to training goals (Russell & Pratt, 1980; Premkumar et al., 2021). Therefore, avatar style and scene context should be calibrated according to users’ anxiety level, training stage, and application goal.

## 7. Conclusions

This study investigated how avatar visual style and environmental context influence user experience and physiological stress responses in VR public speaking scenarios. By integrating subjective evaluations, psychophysiological measures, and qualitative insights, the findings provide a comprehensive understanding of how different design elements shape user responses in immersive social environments.

The results reveal a clear dissociation between subjective experience and physiological stress responses. Natural environments significantly enhanced subjective experience, particularly in terms of satisfaction and overall evaluation, whereas avatar style primarily influenced physiological responses, with stylized virtual audiences eliciting lower electrodermal activity.

Importantly, these findings support a dual-pathway framework in which environmental context shapes cognitive–affective experience, while avatar realism modulates implicit physiological stress responses. This suggests that different components of VR design operate through distinct psychological mechanisms rather than a unified response system.

Contrary to traditional expectations, no significant interaction or mediation effects were observed. Rather than indicating null results, this pattern suggests that physiological responses to avatar realism may be driven by implicit perceptual processes that do not depend on explicit cognitive appraisal.

From a theoretical perspective, this study advances research on social evaluative threat by demonstrating that evaluative cues in VR can differentially influence explicit experience and implicit physiological activation.

From a practical perspective, the findings provide actionable insights for the design of VR training systems. Natural environments can enhance user comfort and engagement, while stylized avatars may reduce stress in socially evaluative contexts such as public speaking. Designing VR systems that balance perceptual realism with psychological comfort may improve both user experience and training effectiveness.

In conclusion, this study highlights the importance of disentangling multiple dimensions of VR design and their underlying mechanisms. Future research should further examine how perceptual realism, environmental context, and interactive feedback jointly influence both subjective and physiological responses, particularly in longitudinal and applied settings.

## Figures and Tables

**Table 1:** Descriptive statistics for subjective and physiological measures (M ± SD) across experimental conditions.

Measure	Natural-Realistic	Natural-Stylized	Indoor-Realistic	Indoor-Stylized	F	*p*	ηp^2^
User Experience (UX)	3.96 ± 0.72	3.90 ± 0.70	3.78 ± 0.74	3.83 ± 0.75	3.85	0.052	0.029
User Satisfaction (US)	3.84 ± 0.79	3.85 ± 0.77	3.62 ± 0.80	3.67 ± 0.82	4.16	**0.043**	0.031
Usability (TU)	3.76 ± 0.80	3.75 ± 0.79	3.64 ± 0.76	3.71 ± 0.78	3.07	0.082	0.023
Perceived Support	3.92 ± 0.68	3.95 ± 0.69	3.55 ± 0.70	3.60 ± 0.72	7.99	**0.006**	0.059
Perceived Threat	2.85 ± 0.89	2.80 ± 0.90	2.98 ± 0.88	2.95 ± 0.91	1.12	0.344	0.012
ΔEDA (μS)	0.75 ± 0.18	0.55 ± 0.12	0.72 ± 0.15	0.54 ± 0.11	7.92	**0.006**	0.058
ΔRMSSD (ms)	−0.33 ± 8.11	0.12 ± 7.95	−1.46 ± 8.49	1.97 ± 8.14	3.11	0.080	0.024

Note. ΔRMSSD = RMSSD_T2 − RMSSD_T1; ΔEDA = EDA_T2 − EDA_T1. Values are presented as mean ± standard deviation. US = user satisfaction; TU = technology usability; UX = user experience. US, TU, perceived audience support, and perceived audience threat were calculated by averaging the corresponding item responses. UX was calculated as the mean of the US and TU composite scores. Bold values indicate statistical significance at *p* < 0.05.

**Table 2:** Results of bootstrap mediation analyses for perceived support and perceived threat.

Model 1: User Experience (UX) as outcome (Mediator: Perceived Support, IV: Scene Type)			
Path	β	SE	95% CI
a: Scene → Support	0.399	0.141	[0.120, 0.678]
b: Support → UX	0.285	0.053	[0.181, 0.389]
c: Total effect (Scene → UX)	0.184	0.093	[−0.000, 0.369]
c′: Direct effect	0.071	0.087	[−0.102, 0.243]
ab: Indirect effect	0.113	—	[0.020, 0.220]
Model 2: User Experience (UX) as outcome (Mediator: Perceived Support, IV: Avatar Style)			
Path	β	SE	95% CI
a: Style → Support	0.197	0.141	[−0.081, 0.475]
b: Support → UX	0.285	0.053	[0.181, 0.389]
c: Total effect (Style → UX)	−0.053	0.094	[−0.238, 0.133]
c′: Direct effect	−0.110	0.085	[−0.278, 0.058]
ab: Indirect effect	0.058	0.043	[−0.013, 0.160]
Model 3: Physiological Stress (ΔEDA) as outcome (Mediator: Perceived Threat)			
Path	β	SE	95% CI
a: Style → Threat	−0.066	0.141	[−0.344, 0.213]
b: Threat → ΔEDA	0.018	0.048	[−0.078, 0.113]
c: Total effect (Style → ΔEDA)	−0.212	0.075	[−0.361, −0.064]
c′: Direct effect	−0.211	0.075	[−0.361, −0.064]
ab: Indirect effect	−0.001	0.008	[−0.024, 0.009]

Note: a = effect of independent variable on mediator; b = effect of mediator on dependent variable; c = total effect; c′ = direct effect; ab = indirect effect; CI = confidence interval.

**Table 3:** Summary of qualitative themes and representative participant responses.

Theme	Description	Representative Quotes
Theme 1: Realistic avatars increase evaluative pressure	Realistic avatars enhance the feeling of being observed and judged, increasing social evaluative threat	“It felt like real people were watching me.” “I felt more nervous when they looked realistic.”
Theme 2: Stylized avatars reduce psychological pressure	Stylized avatars are perceived as less threatening and reduce anxiety	“Cartoon audiences feel less stressful.” “It felt more relaxed and less serious.”
Theme 3: Natural environments support emotional regulation	Natural scenes promote relaxation and reduce perceived pressure compared to indoor environments	“The natural scene made me feel calmer.” “Indoor scenes felt more formal and stressful.”
Theme 4: Limited perceived support due to ambiguous feedback	Lack of clear audience reactions makes it difficult to perceive support	“I couldn’t tell if the audience supported me.” “They didn’t react much, so it felt neutral.”

**Figure A1:**
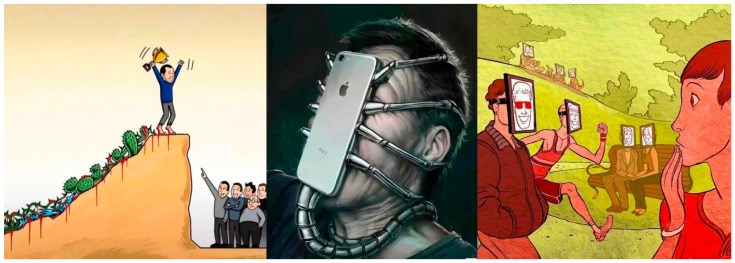
Three images used in the image description task. Participants were instructed to describe the visual content, emotional tone, and underlying meaning of the image, and to provide personal reflections.

## Data Availability

The data presented in this study are available on request from the corresponding author.

## Abbreviations

The following abbreviations are used in this manuscript:

VR	Virtual Reality
UX	User Experience
US	User Satisfaction
TU	Technology Usability
EDA	Electrodermal Activity
GSR	Galvanic Skin Response
HRV	Heart Rate Variability
RMSSD	Root Mean Square of Successive Difference
SDNN	Standard Deviation of Normal-to-Normal Intervals
LF	Low Frequency
HF	High Frequency
ART	Attention Restoration Theory
SRT	Stress Reduction Theory

## Appendix A. Experimental Materials and Questionnaires

This appendix provides the materials used in the VR public speaking tasks, including the speaking topics, image description tasks, and subjective questionnaires administered after the experiment.

### Appendix A.1. Impromptu Speech

Participants were instructed to select one of the following topics for an impromptu speech. They were asked to support their arguments with clear reasoning and concrete examples, and to ensure logical coherence and clarity of expression.

Topic 1. Is environmental protection more dependent on individual behavior or collective policy measures?

Topic 2. Should short-term economic development be prioritized over environmental sustainability?

Topic 3. Should environmental protection rely more on technological innovation or changes in social behavior?

### Appendix A.2. Image Description Task

Participants were presented with an image and instructed to describe its content, emotional tone, and underlying meaning. They were further asked to reflect on their personal experiences and provide their own interpretations and thoughts related to the image.

### Appendix A.3. Subjective Questionaries

Participants completed a set of post-experiment questionnaires to assess user experience, system usability, and perceived audience characteristics. All items were rated on a 5-point Likert scale (1 = strongly disagree, 5 = strongly agree). The questionnaires were adapted from established scales, including the System Acceptance Questionnaire (SAQ), Technology Acceptance Model (TAM), System Usability Scale (SUS), and User Experience Questionnaire (UEQ).

**Table A1:** Subjective Questionaries.

**A3.1. Perceived Usefulness**
PU1. The VR public speaking environment helps improve my speaking ability.
PU2. The VR public speaking environment helps reduce my speaking anxiety.
PU3. The design of the VR environment supports me in completing the speaking task.
**A3.2. Perceived Satisfaction**
PS1. I am satisfied with the overall design of the VR public speaking environment. PS2. The VR environment makes me feel pleasant and relaxed. PS3. I would recommend this VR public speaking training environment to others.
**A3.3. Ease of Use**
EU1. The VR public speaking environment is intuitive and easy to use. EU2. I can quickly learn how to complete tasks in the VR environment. EU3. The functions of the VR system are well integrated, allowing me to complete tasks easily.
**A3.4. Efficiency**
E1. I am satisfied with the response speed of the VR system. E2. I can complete the speaking task within the allotted time. E3. The VR environment effectively improves my speaking efficiency.
**A3.5. Attractiveness**
A1. I am satisfied with the visual appearance of the VR environment. A2. The design style of the VR environment is novel and engaging.
**A3.6. Audience Realism**
AR1. The behavior and expressions of the virtual audience are realistic. AR2. The virtual audience feels like a real audience listening to me. AR3. The audience appears attentive to my speech.
**A3.7. Perceived Audience Support**
PAS1. The audience’s expressions make me feel supported and encouraged. PAS2. The audience appears interested in my speech. PAS3. The audience behaves in a friendly manner.
**A3.8. Perceived Audience Threat**
PAT1. The audience’s expressions make me feel nervous or anxious. PAT2. The audience makes me feel judged or evaluated. PAT3. The presence of the audience makes it difficult for me to focus on my speech.

## Appendix B. Interview Protocol

### Semi-Structured Interview Questions

This appendix presents the semi-structured interview protocol used to collect qualitative data following the VR public speaking experiment. The interview lasted approximately 5–10 min and aimed to capture participants’ subjective experiences, emotional responses, and perceptions of the virtual environment.

Participants were informed that there were no right or wrong answers and were encouraged to freely share their personal experiences. All interviews were audio-recorded for research purposes, and confidentiality was assured.

**Table A2:** Interview Questions.

**B1.1. General Experience of the VR Environment**
1. How would you describe your overall experience in the VR public speaking environment?
Probes: Which aspects felt realistic? Which aspects felt artificial or less convincing?
2. Did the VR environment help or hinder your performance? Probes: How did the visual design (e.g., scene style) influence your attention or emotions? 3. Were there any elements in the VR environment that felt unnatural or distracting? Probes: For example, background, audience, or sound—did anything break your sense of immersion?
**B1.2. Perceptions of the Virtual Audience**
1. How did the virtual audience affect your emotional state? Probes: Did you feel supported, or did the audience increase your nervousness? 2. Did you notice the audience’s movements or behaviors? How did these affect your performance? Probes: Did their reactions help you focus or distract you? 3. Did the visual style of the audience influence your speaking experience? If so, how?
**B1.3. Emotional Responses and Anxiety**
1. How did your level of nervousness in the VR environment compare to speaking in front of a real audience? Probes: Did the VR environment reduce or increase your anxiety? 2. How did you regulate your emotions during the speech? Probes: Did the VR environment or audience design help you feel more relaxed or focused?
**B1.4. Cognitive Load**
1. How difficult was it to complete the speaking task in the VR environment? Probes: Was it easier or more difficult than real-world speaking? 2. Were there moments when it was difficult to concentrate or think clearly? Probes: Did any visual or auditory elements interfere with your attention?
**B1.5. Suggestions for Improvement**
1. What aspects of the VR environment would you improve? Probes: Audience design, background, sound, or task instructions? 2. If you were to use this system again, what additional features would you like to see? Probes: For example, more realistic audience reactions or interactive elements?
**A3.6. Audience Realism**
AR1. The behavior and expressions of the virtual audience are realistic. AR2. The virtual audience feels like a real audience listening to me. AR3. The audience appears attentive to my speech.
